# Can Intrapleural Tissue Plasminogen Activator and Deoxyribonuclease Be Used to Treat Persistent Hemothorax After Robotic Lobectomy?

**DOI:** 10.7759/cureus.73999

**Published:** 2024-11-19

**Authors:** Deepak Dev Vivekanandan, Mena Louis, Lucas N Canaan, Karen Gersch

**Affiliations:** 1 General Surgery, Northeast Georgia Medical Center Gainesville, Gainesville, USA; 2 Surgery, Northeast Georgia Medical Center Gainesville, Gainesville, USA; 3 Cardiothoracic Surgery, Northeast Georgia Medical Center Gainesville, Gainesville, USA

**Keywords:** deoxyribonuclease, intrapleural fibrinolysis, postoperative management, pulmonary lobectomy, retained hemothorax, tissue plasminogen activator (tpa)

## Abstract

Hemothorax is a serious complication following thoracic surgery, often resulting from vessel injury or rib fractures, and is typically managed with chest tube drainage. Persistent or loculated hemothorax, referred to as retained hemothorax, may require more invasive interventions, such as thoracotomy. Although the intrapleural administration of tissue plasminogen activator (tPA) and deoxyribonuclease (DNase) has shown promise in managing pleural infections, its use for hemothorax remains controversial due to bleeding risks. We present a case of a 74-year-old female who developed a retained hemothorax following a robotic left upper lobectomy for lung cancer. Initial chest tube drainage was insufficient, and her high-risk status rendered her unsuitable for further surgery. After a thorough evaluation and obtaining informed consent, intrapleural tPA and DNase were administered, resulting in significant clinical and radiographic improvement without complications. This case suggests that intrapleural tPA and DNase may be a potential alternative to surgery for managing retained hemothorax. Further studies are needed to establish treatment guidelines.

## Introduction

Hemothorax, the accumulation of blood in the pleural space, is a potentially severe complication following thoracic surgery, often due to vessel injury or rib fractures [1]. Traditional management involves chest tube drainage, but in cases of persistent or loculated hemothorax, more invasive interventions like video-assisted thoracoscopic surgery (VATS) or open thoracotomy may be required to remove the clotted blood and decorticate the pleura [2]. Intrapleural tissue plasminogen activator (tPA) and deoxyribonuclease (DNase) have emerged as potential non-surgical alternatives to promote fibrin breakdown and improve drainage in patients with pleural infection [3]. However, their use in hemothorax remains controversial due to concerns about exacerbating bleeding [4]. This report presents a case of a 74-year-old female who developed retained hemothorax following robotic left upper lobectomy, successfully managed with intrapleural tPA and DNase.

## Case presentation

**Figure 1:**
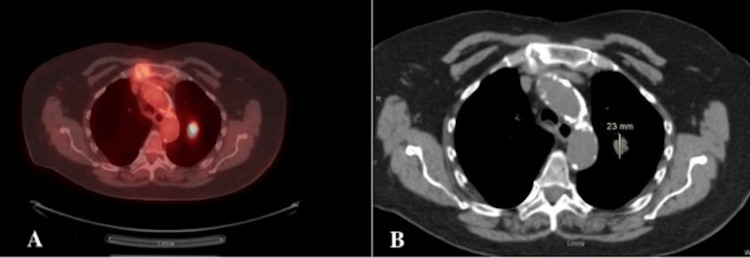
Axial positron emission tomography-computed tomography (PET-CT) scan images. (A) Axial PET-CT scan shows a PET-avid lesion in the left upper lobe of the lung, indicating increased metabolic activity, with a standardized uptake value (SUV) of 7.3, staged as cT1cN0M0 stage IA. (B) Axial non-contrast CT scan of the chest shows a 2.3 cm lesion in the left upper lobe, corresponding to the area of interest seen on the PET-CT.

A 74-year-old female with a history of moderate chronic obstructive pulmonary disease (COPD) and a biopsy confirmed 1.9 cm moderately differentiated adenocarcinoma in the left upper lobe of the lung underwent a robotic left upper lobectomy with lymph node dissection (Figures 1A, 1B).

On the first day postoperatively, the patient had good pain control, with no air leak noted. The chest tube was successfully removed, and she was discharged home in stable condition.

**Figure 2:**
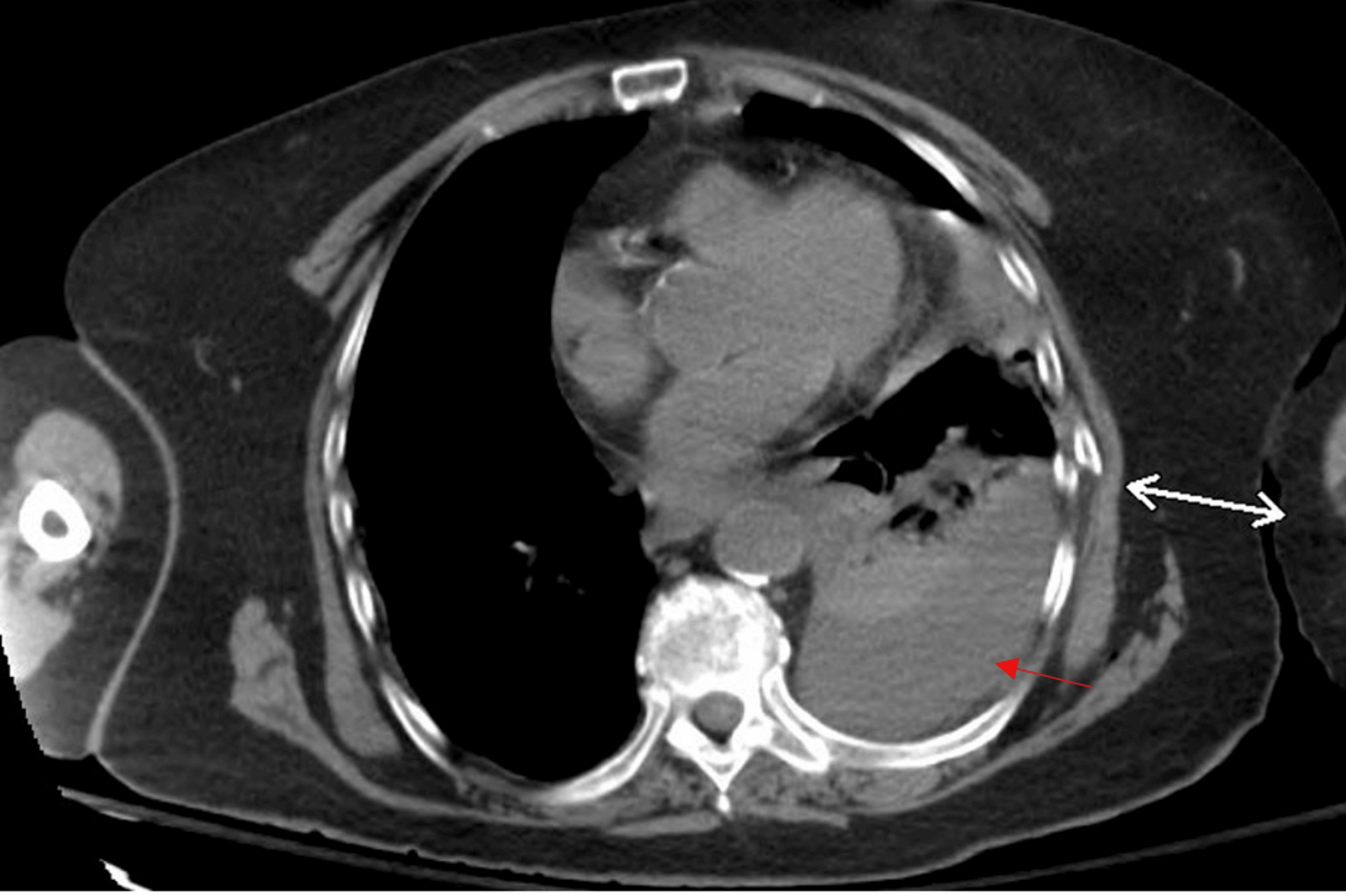
Axial non-contrast CT chest scan. The image shows a left-sided hemothorax (red arrow). A bidirectional arrow highlights the left rib fracture.

On postoperative day two, the patient returned to the emergency department with altered mental status. She was hemodynamically stable. Laboratory investigations showed a decrease in hemoglobin from a preoperative value of 11.8 g/dL to 10 g/dL. A venous blood gas analysis indicated normal partial pressure of carbon dioxide (PCO_2_), ruling out hypercapnia as a cause of her symptoms. A CT scan of the head was negative for acute pathology. A CT scan of the chest revealed a loculated left pleural effusion consistent with hemothorax, as well as multiple left-sided rib fractures (Figure 2). The rib fractures were not present intraoperatively and were likely sustained postoperatively, possibly due to a fall or other trauma during recovery, leading to the development of the hemothorax.

**Figure 3:**
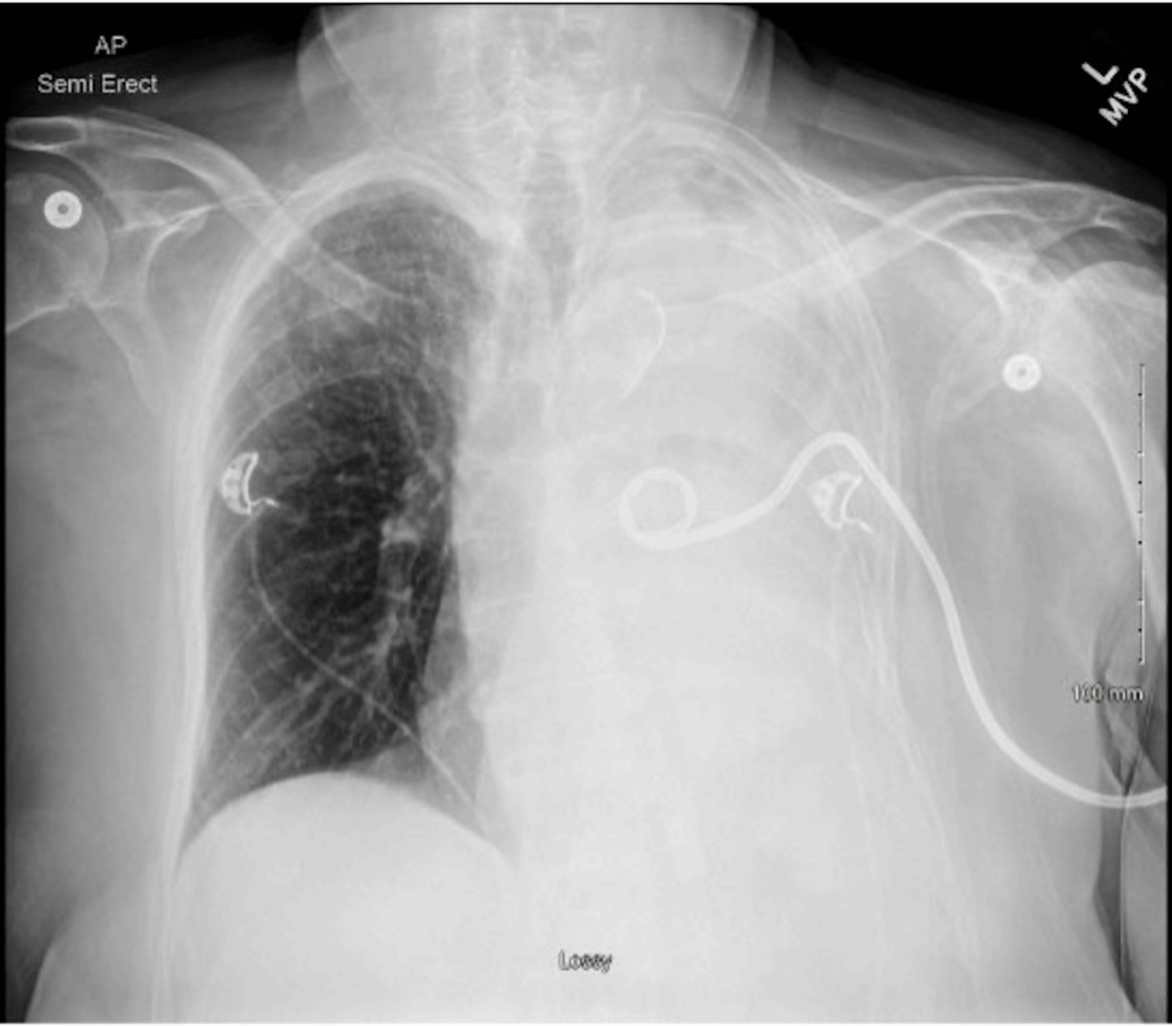
Anteroposterior (AP) chest x-ray showing complete opacification of the left hemithorax. The image indicates a significant pleural fluid accumulation consistent with hemothorax. A left-sided pigtail pleural catheter is also visible, confirming the placement.

On postoperative day three, ultrasound-guided chest tube placement yielded 250 cc of fluid, with minimal improvement on the chest x-ray (Figure 3). By postoperative day four, chest tube output had decreased to 60 cc, but a repeat CT chest scan showed persistent hemothorax with a possible mucus plug. The patient’s mental status continued to improve during this time.

**Figure 4:**
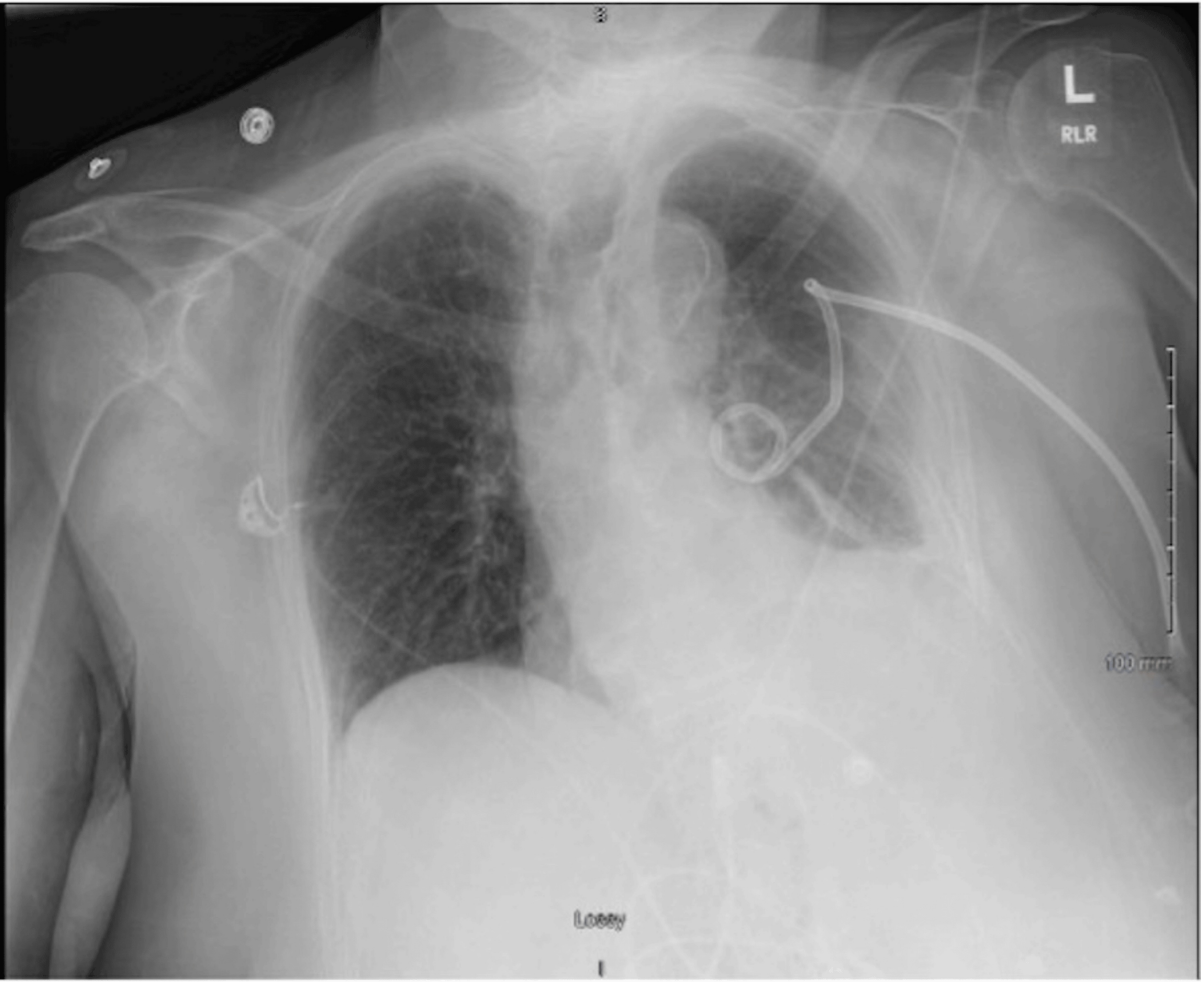
An anteroposterior (AP) chest x-ray. The image shows interval improvement of left hemothorax, with a significant reduction in the opacification of the left hemithorax compared to prior imaging, indicating decreased pleural fluid accumulation. Improved lung aeration is noted, with the left lung demonstrating increased aeration with visible expansion of lung fields, suggesting effective re-expansion of the lung following intrapleural administration of tPA and DNase. The left-sided chest tube remains in situ, continuing to facilitate drainage of the pleural space.

On postoperative day six, a bronchoscopy was performed to remove the left-sided mucus plug, though the subsequent chest x-ray showed no significant change in the hemothorax. With informed consent obtained, a decision was made to initiate intrapleural fibrinolytic therapy. A half-dose of intrapleural tPA and DNase was administered, resulting in a marked improvement in fluid drainage. The patient reported alleviation of respiratory discomfort and was able to breathe more easily. A chest x-ray showed improved aeration of the left lung and reduced pleural fluid collection (Figure 4).

**Figure 5:**
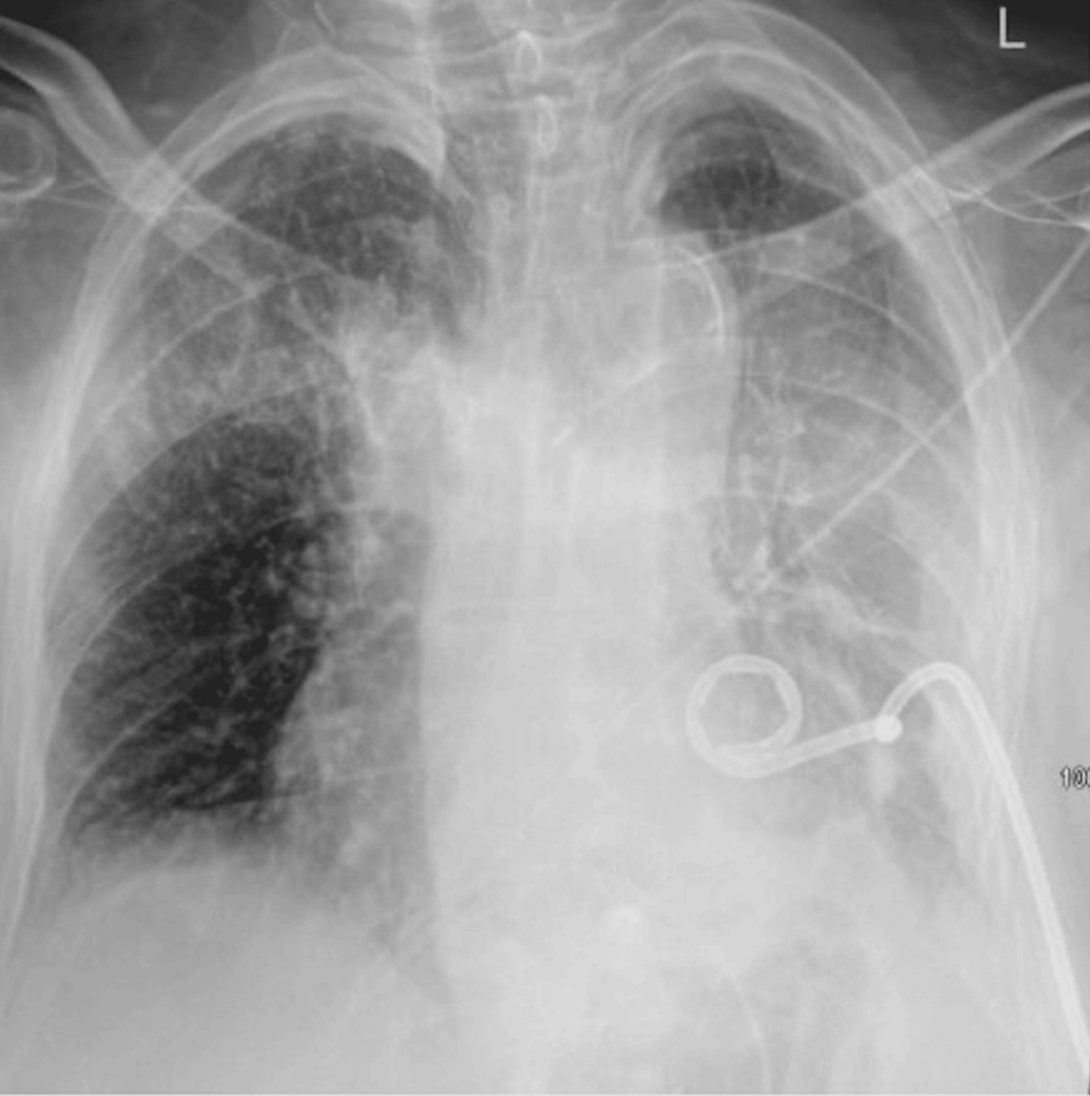
An anteroposterior (AP) chest x-ray demonstrates near-complete resolution of the left hemothorax after intrapleural administration of tPA and DNase. The left lung shows significant re-expansion and improved aeration, now appearing symmetrical to the right lung.

A full dose of intrapleural tPA and DNase was given the following day, leading to further clinical and radiographic improvement. A repeat chest x-ray demonstrated near-complete resolution of the pleural effusion with full re-expansion of the left lung (Figure 5).

Given her stable clinical status and radiographic improvement, the chest tube was removed, and the patient was discharged to a subacute rehabilitation facility for continued recovery and physical therapy. At follow-up appointments, the patient remained asymptomatic with no recurrence of the hemothorax. Chest imaging confirmed sustained resolution of the pleural effusion and full lung expansion. There were no late complications such as infection, bleeding, or fibrothorax.

## Discussion

Hemothorax is a recognized complication following thoracic surgery resection, often resulting from vessel injury or rib fractures [1,5]. The management of retained hemothorax following thoracic surgery poses significant clinical challenges, especially in elderly patients with comorbidities that elevate surgical risks. In this case, the hemothorax was likely secondary to multiple rib fractures. Standard management typically involves chest tube drainage to evacuate blood from the pleural cavity [6]. However, in the presented case, a 74-year-old female developed a retained hemothorax after a robotic left upper lobectomy, which usually necessitates additional interventions, such as video-assisted thoracoscopic surgery (VATS) or thoracotomy for decortication [2,7,8]. In recent years, intrapleural fibrinolytics like tPA and DNase have emerged as potential non-surgical alternatives, particularly for loculated pleural effusions and empyema [9,10]. While intrapleural tPA and DNase have been effective in these conditions, their use in hemothorax remains controversial due to the theoretical risk of exacerbating bleeding by disrupting established clots and hemostasis within the pleural space [4].

In this case, the decision to administer intrapleural tPA and DNase was made after careful consideration of the patient's clinical status and the risks versus benefits of alternative treatments. The patient was hemodynamically stable with no signs of active bleeding, and her hemoglobin levels remained relatively stable after an initial postoperative drop. A half-dose of intrapleural tPA and DNase was administered initially to minimize bleeding risk, leading to improved drainage without a significant drop in hemoglobin. Following this success, a full-dose regimen was given, leading to further clinical and radiographic improvement. Throughout the treatment course, the patient's hemoglobin levels remained stable, and no hemorrhagic events were observed.

The successful outcome in this case suggests that intrapleural administration of tPA and DNase can be a safe and effective alternative to surgical intervention for managing retained hemothorax in selected patients. The theoretical risks associated with fibrinolytic therapy, particularly bleeding, may be mitigated through careful patient selection, dose adjustment, and vigilant monitoring. This approach may be particularly beneficial for patients who are poor surgical candidates due to age, comorbidities, or other risk factors.

While the literature on the use of intrapleural fibrinolytics in hemothorax is limited, existing reports and studies have primarily focused on their efficacy in empyema and complicated parapneumonic effusions [3]. The mechanisms by which intrapleural tPA and DNase facilitate drainage in these conditions are applicable to hemothorax, as both involve the breakdown of fibrinous material that contributes to fluid loculation. The positive results observed in this case align with the underlying pharmacological actions of these agents.

The implications for clinical practice include the potential to expand treatment options for retained hemothorax beyond surgical intervention. Intrapleural fibrinolytic therapy could reduce hospital stays, lower healthcare costs, and improve patient quality of life by avoiding the morbidity associated with additional surgery. However, the decision to use this therapy must be individualized, taking into account the patient's overall health status, stability, and the absence of contraindications such as active bleeding or coagulopathy. Further research is needed to establish standardized guidelines for the use of intrapleural tPA and DNase in hemothorax.

## Conclusions

Intrapleural administration of tissue plasminogen activator (tPA) and deoxyribonuclease (DNase) represents a promising, minimally invasive alternative to surgical intervention for managing retained hemothorax in carefully selected patients. This approach can effectively resolve persistent pleural collections while minimizing the risks associated with additional surgery, particularly in elderly patients or those with significant comorbidities. Clinicians should consider intrapleural fibrinolytic therapy when standard chest tube drainage is insufficient, always weighing the potential benefits against the risks of bleeding and ensuring close patient monitoring. Further studies are warranted to establish standardized guidelines and confirm the safety and efficacy of this treatment modality in a broader patient population.
